# CCHFV GP38 and GP85 interact with cell-surface glycosaminoglycans

**DOI:** 10.1038/s44298-026-00198-1

**Published:** 2026-05-20

**Authors:** Olivier Reynard, Romain R. Vivès, Olga Makshakova, Nosylys Gelas, Estelle Gallice, Anna Papa, Christophe Peyrefitte, Viktor E. Volchkov

**Affiliations:** 1 ImmunoBiology of Viral Infection, CIRI, Centre International de Recherche en Infectiologie, INSERM U1111, CNRS, UMR5308, Univ Lyon, Université Claude Bernard Lyon, Lyon, France; 2 Molecular Basis of Viral Pathogenicity, Centre International de Recherche en Infectiologie (CIRI), INSERMU1111-CNRS UMR5308, Université Claude Bernard Lyon 1, Lyon, France; 3 CNRS, CEA, IBS, Univ. Grenoble Alpes, Grenoble, France; 4 Faculty of Biology, Univ. Grenoble Alpes, Signalling Research Centres BIOSS and CIBSS, Synthetic Biology of Signalling Processes Lab, University of Freiburg, Freiburg, Germany; 5 Department of Microbiology, Medical School, Aristotle University of Thessaloniki, Thessaloniki, Greece; 6 Institut Pasteur de la Guyane, Cayenne, France; 7 Institut Pasteur, Paris, France; 8 UA-17-INSERM, Sante des Populations en Amazonie, Cayenne, France

**Keywords:** Biochemistry, Microbiology, Molecular biology, Structural biology

## Abstract

Crimean Congo Hemorrhagic Fever Virus (CCHFV) is a negative-strand segmented RNA virus responsible for severe hemorrhagic fever in humans. The M genomic segment of CCHFV encodes a polyprotein precursor that is processed by cellular proteases into several structural and non-structural proteins. Among them, GP38 and its precursor GP85 are known to be secreted into the extracellular environment. We investigated their abilities to bind cells and we identified that they strongly bind to the cell plasma membrane through interaction with glycosaminoglycans (GAGs). This interaction was mapped to a surface-exposed basic cluster that combines both a prototypical GAG-binding domain and linearly distant amino acids. The present study describes for the first time the interaction between CCHFV GP38/85 proteins and host cell GAGs and characterizes the interaction domain.

## Introduction

Crimean Congo hemorrhagic fever virus (CCHFV) was discovered in 1944 and was identified as being the causative agent of severe hemorrhagic fever^1^. This virus belongs to the order *Bunyaviridae*, family *Nairoviridae*, genus *Orthonairovirus*. CCHFV is an arbovirus hosted by ticks, notably those from the genus *Hyalomma*. The geographical distribution of CCHFV overlaps the distribution area of those hard ticks that range from Africa, Asia, central Europe, eastern Mediterranean countries (Balkan countries and Turkey), and more recently Western European countries e.g., Spain and France^2–4^. The first symptoms arise 5–15 days after initial infection and consist of an acute febrile illness, which is followed by a severe phase generally including hemorrhagic symptoms and characterized by low platelet count, coagulopathy, acute liver infection, and the release of high amounts of pro-inflammatory mediators^2^. The disease leads to death in up to 30% of the cases^5,6^.

The genome of CCHFV is composed of three segments termed large (L), medium (M), and small (S), which respectively encode for the RNA polymerase, the glycoproteins, and the nucleoprotein as well as in an ambisense orientation the non-structural proteins S and NSs^7^. The glycoproteins are processed co-translationally by cellular proteases, notably furin and SKI-1 proteases^8,9^. This proteolysis and the complex O- and N- glycosylations lead to the generation of three predicted non-structural glycoproteins: the mucin-like domain (MLD), GP38, and NSm, as well as two structural glycoproteins Gn and Gc with the later being involved in both low-density lipoprotein receptor (LDLR) recognition and viral envelope fusion after endocytosis^8,10–13^. The MLD is a highly glycosylated disordered protein that contains a high amount of O-linked glycans and five N-glycans. MLD and GP38 proteins (also named GP1) were characterized or predicted in several nairoviruses, including CCHFV and Dugbe virus^7^. While the MLD sequence is of low amino-acid homology between CCHFV isolates, GP38, Gn, NsM, and Gc are more conserved^7^.

In the supernatant of cells transfected to express the M segment or infected with CCHFV, GP38 is mostly found in a free form, but also as a non-furin-digested precursor named GP85 containing both GP38 and the MLD^8^. In addition, Western blot analysis of supernatants shows the presence of another, poorly characterized ~160 kDa protein, comprising both GP38 and MLD^8^. Although the functional role of GP38 during CCHFV infection remains unclear, monoclonal antibodies directed against GP38 protect non-human primates from CCHFV lethal challenge and a vaccine targeting GP38 can protect against lethal infection, thus indicating that GP38 is a critical pathogenicity factor^14,15^. Glycosaminoglycans (GAGs) are targeted by a wide range of pathogens including bacteria, parasites, and viruses^16–20^. This observation was initially documented in 1966 in a rabbit model of HSV1 infection in which heparin was shown to protect from infection^21^. Since then, GAGs are documented as a co-factor of infection for multiple viruses, including the more recent SARS-COV-2^22^. Here, we show that secreted GP38 and GP85 bind cell surface GAGs and we identify the precise binding domain.

## Results

### GP38/85 binds to the cell surface

**Fig. 1:**
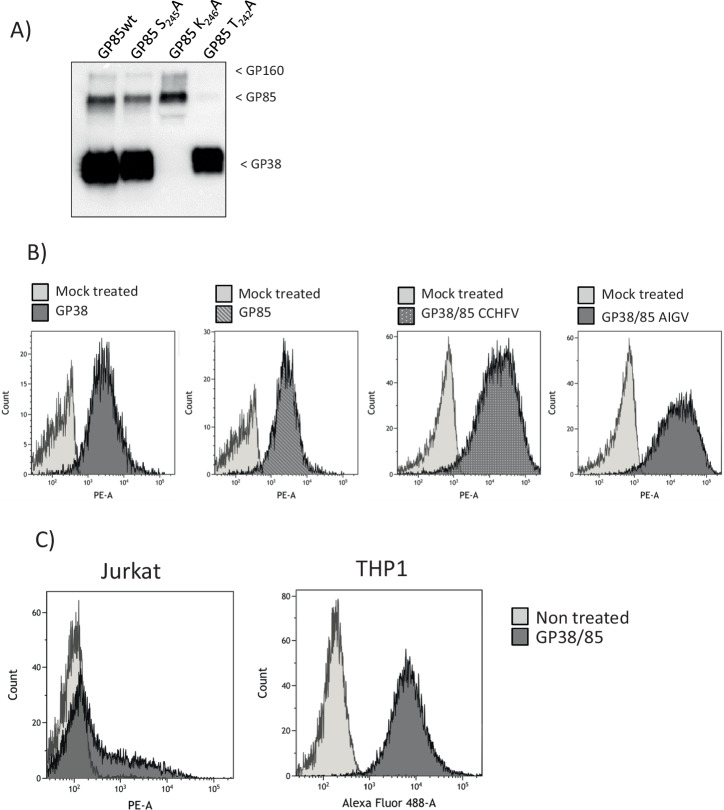
GP38 and GP85 bind to cells through GAGs. **A** Analysis of GP38/85 expression profile in the supernatant of HEK293T cells transfected with 6xHis tagged GP85 expression vector (wt or T242A, S245A, K246A mutant) harvested at 48 h post transfection. Western blot was revealed using an anti-6xHis tag HRP antibody. **B** Binding of GP38 or GP85 only and GP38/85 derived from CCHFV sequence (Ibar10200) or Aigai virus GP38/85 to HEK293T after incubation on ice and subsequent analysis by flow cytometry on a BS LSR4, detection using anti His-PE. **C** Binding of GP38/85 to Jurkat or THP-1 cells as described above.

**Fig. 2:**
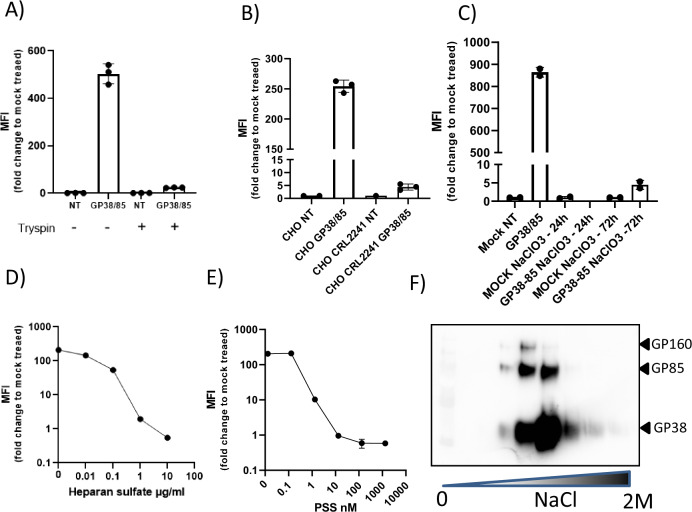
GP38/85 binds glycosaminoglycans. **A** HEK293T cells treated by 0.25% trypsin for 5 min at 37 °C and further subjected to GP38/85 binding assay and analyzed by flow cytometry. PE-mean fluorescent intensity (MFI) was normalized to the mock treated condition. **B** Wildtype CHO cells or CHO cells deficient in GAG synthesis were subjected to GP38/85 binding assay. **C** HEK293T cells were cultured in the presence of NaClO3 at 30 mM for 24 h or 72 h (24 h then 48 h with medium renewal) and then subjected to GP38/85 binding assay and analyzed by flow cytometry. **D**, **E** Binding of GP38/85 on HEK293T in the presence of increasing concentration of heparan sulfate (**D**) or poly(4-styrene sulfonic acid) 75 kDa polymer (PSS, E). **F** Strength of GP38/85—GAG interaction was evaluated through the binding of proteins to Heparin sepharose and further elution with a NaCl gradient. Fractions were analyzed by Western blot and anti-6xHis HRP antibody. **E**, **F** The experiment displayed is the representative experiment of a biological triplicate, each done in technical triplicate.

Research conducted to date has shown that cells infected with CCHFV release GP38/85 into the culture medium^8^. In the current study, we investigated the cell-binding properties of GP38. The GP38 protein is produced from the GP85 precursor by furin cleavage between MLD and GP38. In order to produce GP85 only, we mutated lysine 246, a key basic residue of the furin cleavage site. This mutation led to the abrogation of cleavage and sole GP85 release (Fig. 1A)^8^. Furin cleavage sites are known to be sensistive to neighboring O-glycans for efficient cleavage^23,24^, we therefore mutated in GP85 expressing plasmid potential O-glycosylation sites: threonine 242 and serine 245. As shown in Fig. 1A, mutation of residue at position 242 led to the disappearance of GP85 precursor leading to GP38 protein only, while S245A mutation did not affect protein processing. To assess the cell-binding capacity of these proteins, we incubated on ice, 6xHis tag-purified GP38 or GP85 (strain Ibar10200) with HEK293T cells and monitored the presence of cell-surface bound protein by flow cytometry using anti-His antibody. We observed a strong binding of both GP38 and GP85 to the whole cell population (Fig. 1B). The specificity of the assay was confirmed by the absence of interaction of an unrelated 6xHis tagged protein (VSV-N, Supplementary Fig. 1C). We also observed a similar binding level using a GP38/85 originating from Aigai Virus (AIGV, former CCHFV genogroup VI, Europe 2 genotype, AP92 strain^25^), a virus closely related to CCHFV (Fig. 1B, sequence alignment in Supplementary Fig. 1D). This ability to bind cell surface was identified in several cell lines including monocytic or lymphoid cells (THP1 and Jurkat, Fig. 1C, and CHO Fig. 2C).

To determine the binding mechanism of GP38 to the cell surface, we treated HEK293T cells with trypsin prior to the addition of GP38/85 in order to digest a putative cell-surface GP38/85 protein receptor. As displayed in Fig. 2A, such treatment diminished GP38/85 binding. As trypsin cleaves cell-surface proteins, it simultaneously removes moieties associated with these proteins, including GAGs. These molecules, notably heparan sulfate (HS), are complex negatively charged sulfated polysaccharides with broad biological properties^26^ which are widely used by viruses as cell-surface attachment sites^27,28^. We used CRL-2241 CHO cells, a galactosyltransferase I-deficient cell line unable to perform GAG synthesis to evaluate GP38 binding^29^. As shown in Fig. 2B, those cells exhibited a very limited ability to bind GP38/85 in comparison to WT CHO cells. The binding of proteins to GAGs often relies on interaction of positively charged amino acids with negative charge of GAGs^30^, we then treated cells with sodium chlorate, a known inhibitor of GAG sulfation^31^. After 24 h of treatment with chlorate, the binding of GP38/85 to the cell surface was reduced by 2 logs, and repeated treatment over 72 h fully abbrogated GP38/85 binding (Fig. 2C). Furthermore, we conducted an assay to evaluate the competition with free ligands. Firstly, soluble HS was used as a free competitive ligand and demonstrated a strong binding inhibition with effects seen at a concentration as low as 0.1 µg/mL HS (Fig. 2D). To confirm that this interaction relies on negative charges, we used a polymer of the poly(4-styrene sulfonic acid) (PSS, 75 kDa form) known to be a chemical structural analog of heparin and mimicking heparin negative charges by sulfonate moieties instead sulfates ones^32^. These molecules strongly reduced GP38/85 binding to the cells at concentration as low as 1 nM (Fig. 2E). The result indicated that binding of GAGs to GP38/85 is dependent on negative charges. Altogether, these results identified GAGs as an efficient cell-surface attachment receptor for GP38 and GP85. GAG-binding proteins are differently sensitive to NaCl concentration depending on their affinity for the polysaccharide^33^. To determine the strength of the GAG-GP38/85 interaction, the proteins were loaded onto a Heparin (HP) sepharose column equilibrated in a 0.1 M NaCl-containing buffer, and the proteins were eluted using a 0.1–2 M NaCl gradient. As shown in Fig. 2F, GP38/85 eluted at 0.5 M NaCl indicating a mid-strength interaction. In addition, all forms of GP38/85 (GP160) were bound to the column and eluted in the same fractions confirming the results of Fig. 1B.

**Fig. 3:**
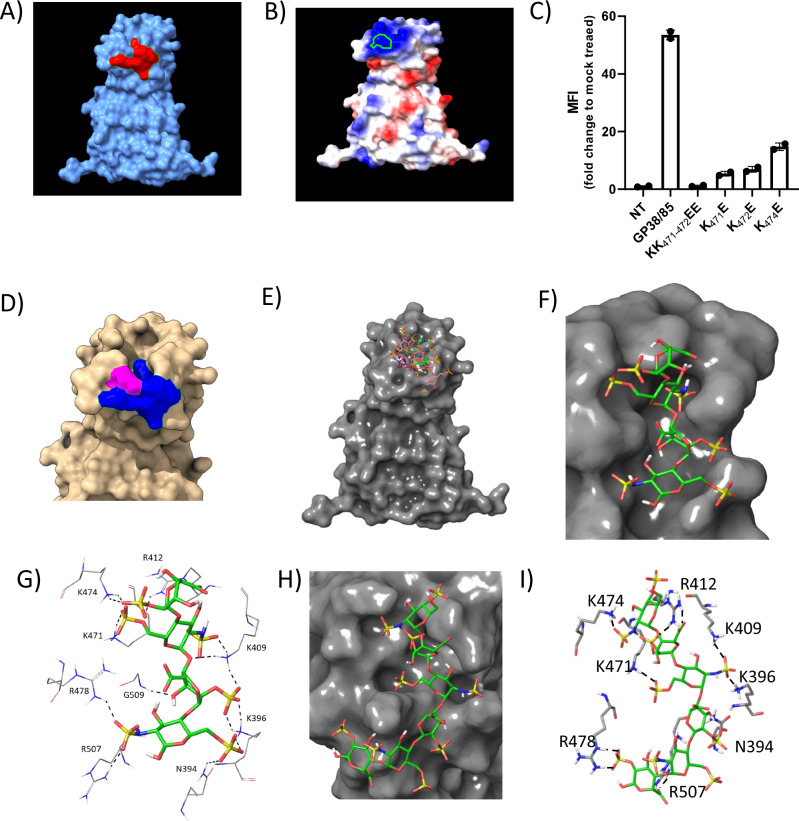
Identification of the GAG-binding domain. Critical amino acids for GAG-binding were mapped on the structure of GP38 (PDB 6VKF) using Chimera software. **A** Cardin-Weintraub motif amino acids colored in red, **B** electrostatic coloring on cell surface, position of arginine 412 is highlighted in green. **C** Individual mutants of the -BBXB- motif were produced, purified, and further assayed in cell binding assay, all proteins were assayed at 1 µg/mL. **D** Position of arginine 412 on the GP38 3D structure. **E** Surface view of the most favorable binding position of a tetrameric heparan sulfate fragment determined by in silico docking. **F** Detailed view of the HS fragment in the predicted binding canyon. **G** The scheme of closest contacts in the GP38–heparin complex (color code: C atoms – gray for protein and green for heparin sulfate, O atoms – red, N – nitrogen, S – yellow, polar H – white, non-polar H are not shown for clarity). **H** Most energy favorable complex of GP38 with hexameric HS determined by molecular dynamics (MD) simulation. **I** Scheme of the closest contacts in the GP38– hexameric HS complex determined by MD.

As mentioned above, GAG binding to proteins often relies on the interaction with basic residues. These basic residues are often organized as clusters on 3D structure, or even be linearly grouped as described in the prototypic Cardin-Weintraub (C-W motif) GAG binding motif -BBXB- (where B is a basic motif amino acid and X a random amino acid)^34^. The analysis of the GP38 sequence revealed a cluster of basic residues between positions 471 and 474, which fitted the Cardin-Weintraub motif (-KKNK-). This motif is conserved within all CCHFV strains including the most distant AIGV. The mapping of this motif on the atomic structure of GP38^35^, showed that these residues are not randomly distributed on the protein structure but exposed at the surface of the protein and clustered to form a defined positively charged area (Fig. 3A, B), thus supporting a potential role as a GAG-binding domain. Basic residues of the C-W motif were mutated to glutamic acid and all mutants were produced and purified from HEK293T supernatants (Supplementary Fig. 1A). In the C-W motif, the single mutation of each basic residue resulted in the loss of GP38’s ability to bind to 293T cells (Fig. 3C).

**Fig. 4:**
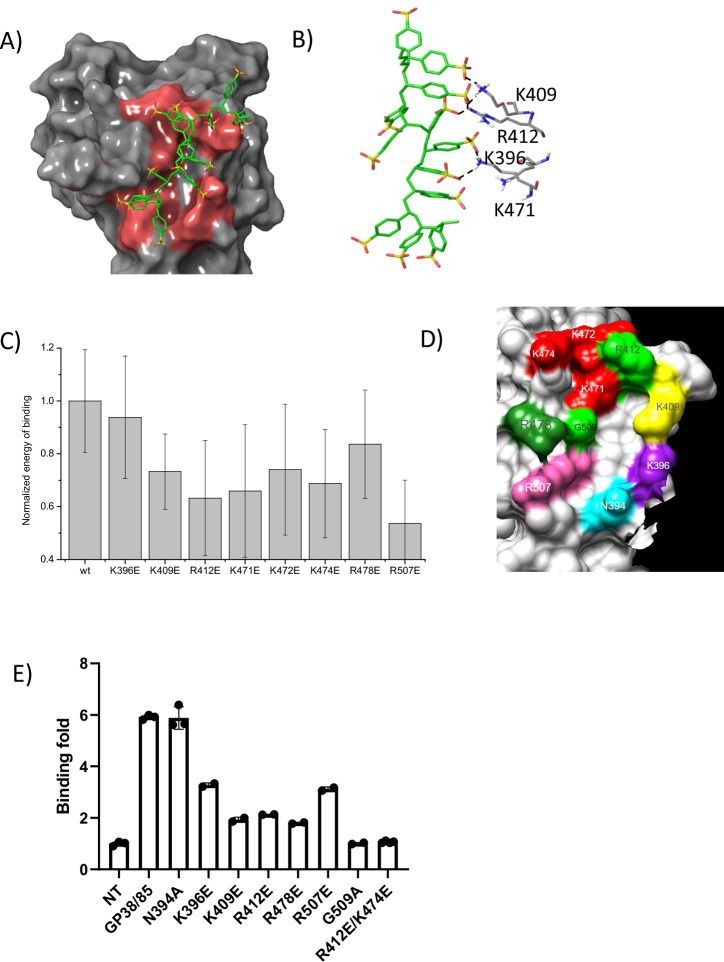
Characterization of the GAG binding domain. **A** Analysis by molecular dynamics of the PSS interaction with GP38, a detailed view of the PSS molecule in the predicted binding canyon. **B** The scheme of closest contacts in the GP38–heparin complex (color code: C atoms – gray for protein and green for heparin sulfate, O atoms – red, N – nitrogen, S – yellow, polar H – white, non-polar H are not shown for clarity). **C** Impact of the mutation of GAG binding associated residues predicted by MD. **D** Critical amino acids for GAG-binding identified by structure analysis and MD were mapped on the structure of GP38. **E** Individual mutants were produced, purified, and further assayed in cell binding assay, all proteins were assayed at 1 µg/mL.

It is interesting to note that the GP38 3D structure demonstrates that the -BBxB- motif is the entry point to a canyon that includes arginine 412. The crests on both sides feature predominantly basic residues, suggesting that the C-W motif may not be solely responsible for GAG binding. (Fig. 3D). We characterized HS binding to GP38 using in silico analysis. We performed fragment-based molecular docking in “blind” mode, when a short fragment of HS (tetramer) was allowed to interact with the whole protein surface. Results revealed only one binding site, which was located in the canyon opening at the -BBxB- motif (Fig. 3E). The tetramer in the most stable position (Fig. 3F) showed interaction with several positively charged residues (Fig. 3G). Then, molecular dynamics (MD) simulations were performed to equilibrate the complex in the presence of explicit solvent (Supplementary Fig. 2A, B). For this, we used a hexameric HS fragment, which had the advantages to be as long as the binding canyon estimated by docking, and was of comparable length to many the GAG fragments length interacting with other proteins^36^. Starting from different HS orientations in respect to the protein we demonstrated the preference of SO_3_^–^ group at N-2 position of one of N-sulfated glucosamine residues to be directed to K471 and K474 (Fig. 3H shows the most energy favorable complex with HS, other possible binding modes are given in Supplementary Fig. 2C–F, the per-residue contacts with HS in the course of trajectory are listed in Table S1). The PSS used in Fig. 2F was also evaluated in the MD simulation and shows that this molecule predominantly occupies the GAG-binding site and interacts with the same basic residues that HS (Fig. 4A, B). Both HS and PSS interact not only with the C-W motif but also with several other positively charged residues. In silico mutagenesis was performed and revealed that the residues K409, K396, R412, K471, K472, K474, R478, and R507 strongly contribute to the HS binding (Fig. 4C), confirming our initial findings in Fig. 3C and expanding the number of potential interacting residues in the GAG binding domain.

To confirm in silico analysis, we performed an additional set of amino acids mutations located either downstream (N394, K396, R412, K409) or upstream (R478, R507, and G509, supplementary Fig. 1B) of the -BBxB- motif amino acids and forming the binding pocket to determine their involvement in the GAGs binding ability of GP38/85 (Fig. 4D). All but N394A impaired cell attachment phenotype (Fig. 4E). The dual mutation of R412 and K474 that maps the entry of the canyon fully abrogated binding of GP38 to cells and therefore confirmed predictions of molecular docking. During viral glycoproteins maturation, GP38 interacts with Gn but later dissociates to be released, as illustrated in supplementary Fig. 3, and GAG binding domain is not involved in this specific interaction^37^.

## Discussion

GP38 function in viral cycle and its potential role in CCHFV pathology has started to be unveiled, notably with the identification of protective GP38-specific antibodies and the involvement of GP38 in endothelial cells damages^14,38^. A recent study has highlighted that human survivors produce protective anti-GP38 antibodies which underline the importance of this protein into viral pathogenesis^39^. Among the available structures of GP38 in the interaction with an antibody, none of them interacts with the GAG-binding cavity (ADI-46143, -46152, -58048, -63530, C13G8 and CC5-17), even one of them might contact Lysine 474 (ADI-63530)^39,40^.

In the current study, we characterized the attachment of CCHFV GP38 to cells and identified GAGs as cell-surface attachment receptor. We mapped the interacting domain on GP38 3D structure both using in silico prediction and mutagenesis. To produce GP38 alone, we kept initial construct containing MLD and we increased its processing by furin through removing inhibition due to O-glycosylation of threonine 242 residue. O-glycosylations are due to O-GalNac transferases that comprise up to 20 members that are either ubiquitously expressed or cell-type specific^41^. Future studies might investigate the nature of the O-GalNac transferase involved in T242 to better understand GP85 secretion. Indeed, we demonstrated that GP85, as it contains GP38, binds to surface GAGs, which may decorate the cell surface with the mucin-like domain. GAGs are long polyanionic sulfated polysaccharide structures that are anchored on proteins present in intracellular compartments and cell surfaces^42^.

In the present study we have demonstrated by different methods that GP38 binds to cell surface GAGs. This binding showed different intensity depending cell type which could be linked to differential amount of heparan sulfates between cell lines (^43^ and supplementary Fig. S4).

GAG-binding domains are often composed of basic amino acids (lysine, arginine) that exhibit positive charges supposed to interact with negative charges carried by the GAGs. In our study both HS and PSS showed activity in reducing GP38 binding indicating that charge either from sulfate or sulfonate moieties are important for interaction. Of note, very similar compound to PSS, Tolevamer has been tested in two phase II clinical trials against *Clostridium difficile* without significant toxicity^44^, suggesting that such a molecule could potentially serve as a basis for antiviral development.

In GP38, the positive amino acids cluster is composed of both a prototypical Cardin-Weintraub GAG-binding domain (XBBXBX, where B are basic amino acids) and a set of surface-exposed amino acids aligned on both crests of a cleft. Interestingly, among the Genbank accessible sequences of the CCHFV M segment, the prototypical binding domain xBBXBX is conserved in all sequences except in one isolate from 1968 (isolate Taf,^45^). It is worth noting that one non-positively charged residue, G509, was found to be important for ligand binding. This may be due to the fact that the mutation had affected the functional positioning of some basic residues, directly involved into complex formation.

The experimental data from this study are fully corroborated by the in silico analysis which demonstrates that the unique docking site of HS is the one identified by our mutagenesis screen. MDs simulations were run using different initial orientations of the HS fragment in respect to the protein’s binding site, and have demonstrated in all tested simulations the importance of N-sulfated moieties for binding. Hundreds of proteins are known to possess GAG-binding domains that modulate their function, including proteins involved in coagulation (antithrombin), immune response (CCL21, IL-8), and growth factors/receptor signal transduction complexed (VEGF/VEGFR, FGF/FGFR). VEGF plays an important role in endothelium homeostasis ensuring notably endothelial cells survival. As VEGF also contains a GAG binding domain, we may speculate a competition for GAG interaction between VEGF and GP38, resulting in altered signaling and further on altered endothelium homeostasis^46^. Therefore, the potential activity of CCHFV GP38/85 within those pathways should be further investigated to identify GP38/85 function in the viral cycle^33,47–49^. As GP38 is known to be present on cell surface as well as virion envelope, GAG interaction with virion-associated GP38 might help increasing the attachment of viral particles to the surface of target cells^14,50^. Soluble GP38/85 may saturate local GAGs, limiting binding of viral particles, to enhance more distant viral spread. Whether GP38 is bound to virion through GAG interaction or an interaction with Gn remains to be elucidated. In addition, little is known about the GAG biology in ticks, and therefore, the role of GP38 in its natural host is even more obscure. However, several tick proteins have been described to possess GAG binding properties, indicating that such motifs exist in ticks, as described for other arthropods^51–54^. Overall, our study sheds light on the mechanism by which the CCHFV GP38/85 proteins bind to the surface of cells, which could improve our understanding of the viral cycle and facilitate the development of viral inhibitors.

## Methods

### Cells, reagents, and transfections

HEK293T cells were cultured in Dulbecco’s modified Eagle’s medium Glutamax (Thermo) supplemented with 10% fetal calf serum (FCS) and Normocin (Invivogen) at 37 °C. Jurkat, THP1 and total lymphocytes were cultured in RPMI Glutamax (Thermo) 10% FCS. Cell transfections were carried out in accordance with the manufacturer’s instructions using the Jet Optimus (Polyplus) reagent at a ratio of 1:1 (plasmid:reagent).

### Plasmids and mutagenesis

The GP85 sequence was subcloned from plasmid pCDNA3.1 M Ibar10200 (Genbank AF467768.2) into vector phCMV with a 6xHis tag at the carboxy terminus. Aigai virus (AIGV, former CCHFV genogroup VI, AP92 strain^25^) M segment (Genbank DQ211625.1) was amplified by RT-PCR from RNA extracted from the supernatant of infected cells and GP85 sequence was further subcloned into phCMV vector with a 6xHis tag at the carboxy terminus. Mutagenesis of GP38 expression plasmid was performed using the Q5 mutagenesis kit (New England Biolabs) according to the manufacturer’s instructions.

### Protein production and purification

Culture medium from phCMV GP85-6xHis transfected HEK 293Tcells was harvested 48–72 h post-transfection, clarified by centrifugation (3000 × *g*, 5 min), and filtered through 0.22 µm filter (Millipore). Tetradentate chelating agarose resin with divalent cobalt (Co2+) (Thermo) was used for the affinity purification step. Binding was performed in the presence of 5 mM imidazole. The resin was washed twice with a 30-bed volume of PBS 10 mM Imidazole. Elution was performed by applying a 200 mM Imidazole solution in PBS pH 7.5. Proteins were further dialyzed against PBS in 5 kDa cartridge (Thermo). The purified proteins contain an average of 85% of GP38, 12% of GP85 (precursor), and 3% of GP160. The 6xHis tagged VSV-N protein used as a control in supplementary Fig. 1D was a kind gift of Dr Louis-Marie Bloyet and Serigne Saliou-Mbacke-Ndyaie.

### Flow cytometry analysis of GP38/85 binding

The binding assay was performed using 105 cells per condition. These were incubated on ice for 5 min, then spun for 1 min at 750 × *g* and incubated with purified GP38/85 (and VSV-N) proteins (10 µg/mL) for 30 min on ice. Cells were then washed twice with ice-cold PBS and stained using an anti 6xHis antibody labeled with phycoerythrin (Miltenyi biotech) for 20 min on ice. Stained cells were washed twice with ice-cold PBS and analyzed directly on an LSR Fortessa 5 L flow cytometer (Becton Dickinson). Heparan Sulfate expression at the cell surface was assessed by flow cytometry. Adherent CHO cells were rinsed with PBS and detached using Accutase (ThermoFisher). Both adherent (CHO) and non-adherent (Jurkat) cells were washed once with FACS buffer (PBS complemented with 1% BSA and 1 mM EDTA) and incubated with 10E4 primary antibody (1:200 dilution in FACS buffer) for 1 h at 4 °C. Cells were washed twice and then incubated with the anti-mouse-cyanine 3 secondary antibody (1:200 dilution in FACS buffer) for 1 h at 4 °C. After washing twice, cells were fixed with 2% paraformaldehyde in PBS for 10 min at 4 °C. Data were analyzed using flow cytometer (MACS Miltenyi Biotec) and processed through its associated software, MACSQuantify.

### In silico simulations

To identify the location of heparin-binding site, the molecular docking of heparin to GP38 was performed using ClusPro web server^55^. Then, complexes of GP38 and heparin were equilibrated using MDs simulations to take explicitly into account both ligand and protein flexibility in water solvent. The hexamer of heparan sulfate was placed in proximity of the binding site, determined by molecular docking, and at the distance ~5 Å from the protein surface to allow both protein and polysaccharide to adjust their geometries in the course of the complex formation. MDs simulations were carried out in Amber 18 package^56^, GLYCAM06j force field was used for heparin^57^, generalized amber force field (gaff) for the chemical analog of GAG^58^, and AMBER14SB for protein molecule^59^. The in silico site-directed mutagenesis was performed based on above described structures of native GP38 and HS complexes via a replacement of a target basic residue by glutamic acid. The new structures GP38 mutants with HS bound were equilibrated in the course of 400 ns MD trajectory.

### Sodium chlorate treatment

In order to inhibit GAGs sulfation, HEK293T cells were treated for 24 or 72 h with 30 mM sodium chlorate in DMEM (3% FCS). The 72 h treatment was performed as an initial 48 h treatment followed by medium renewal and a second 24-h incubation with 30 mM sodium chlorate in DMEM3% FCS. The cells were then assessed for GP38/85 binding by flow cytometry.

### Heparin-binding analysis

The His-tagged purified proteins were diluted ten times in 50 mM Tris pH 7, 1 mM NaCl, and then loaded onto a Heparin column (GE Healthcare). The column was washed with three column volumes of binding buffer. Repeated elutions were performed with increasing concentrations of NaCl ranging from 0.1 to 2 M. The fractions were analyzed by Western blot using an anti-His HRP conjugated antibody (Miltenyi Biotech).

## Supplementary information

Supplementary information

## Data Availability

The datasets generated and/or analyzed during the current study are not publicly to prevent unnecessary carbon-emitting cloud storage but remain available from the corresponding author on reasonable request. Sequences: NC_078227.1, AF467768.2 and structures: PDB 9B8J, 6VKF were used in the current study.

## Code availability

Kaluza software version 2.3.00000.20268 and MacsQuantify software were used for flow cytometry. Molecular docking was perfomed using the ClusPro web server. Molecular dynamics was done using Amber18 package with GLYCAM06j force field used for heparin, generalized amber force field (Gaff) for the chemical analog of GAG, and AMBER14SB for the protein molecule.
