# Supplementary material for: CCHFV GP38 and GP85 interact with cell-surface glycosaminoglycans
Source: Npj Viruses. 2026 May 20;4:37. doi: 10.1038/s44298-026-00198-1 (PMC13458711; doi:10.1038/s44298-026-00198-1)
Supplement: Supplementary file 1 [file 44298_2026_198_MOESM1_ESM.pdf]

Supplemental information

Supplementary figure 1

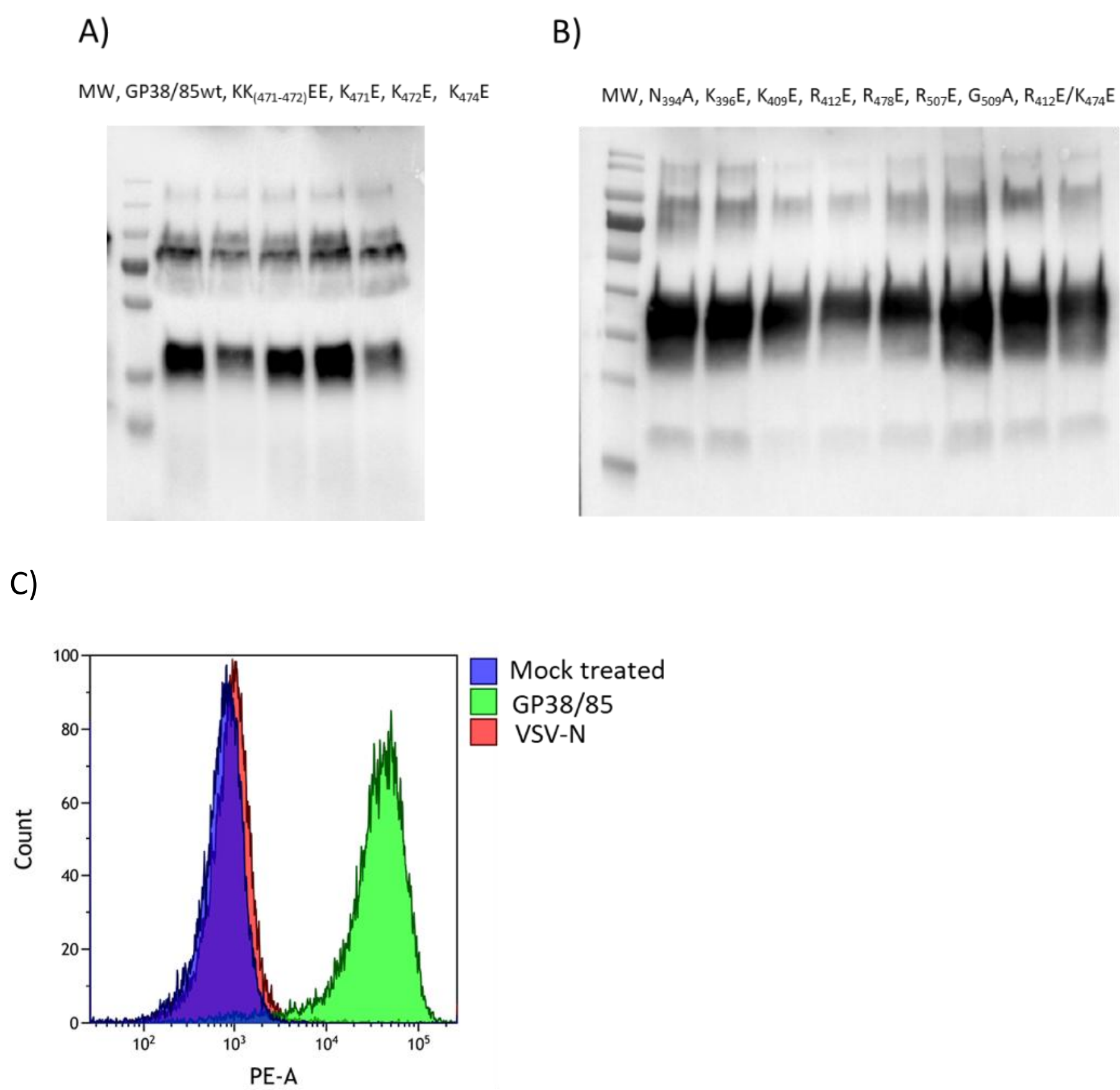

D)

```
CCHF            M--H---ISLMYAIL---------CLQLCG-LGETHGSHNETRHNKTDTMTTPGDNPSSE
Aigai           MPDHFILLLLMYTVFHQHPQDVSANVHLVNPSNSTTGA--STAFNPVHNATTDGQKQ---
                *  *   : ***:::          ::* .  ..* *:  .* .* ... ** *::

CCHF            PPVSTALSITLDPSTVTPTTPASGLE--GSGEVYTSPPIT-TGSLPLSETTPELPVTTGT
Aigai           ---STSTTTMARPSTVTQITTAQAMEVDGSGESTTLPAVTPTPAETTTHANQKSASTMGT
                   **: :    *****  *.*..:*  ****  * *.:* * : . :.:. : . * **

CCHF            DTLSAGDVDPSTQTAGGTSAPTVRTSLPNSPSTPSTPQDTHHPVRNLLS-VTSPGPDETS
Aigai           LTTSLEDTALMTLTGRSILTSQSQTGSFDRTSKPQTPRSTNQPLRKLLSAVSTEAAPQTS
                 * *  *.   * *. .  :.  :*.  : .*.*.**:.*::*:*:*** *:: .. :**

CCHF            TPSGTGKESSATSSPHPVSNRPPTPPATAQGPTENDSHNATEHPESLTQSATPGLMTSPT
Aigai           PTAVTADQTEASTSPQVTTDTSPSTPTQVTSSAGSSSSNTSNHSKTTPQLTSSGSEAQQT
                ..: *..::.*::**: .:: .*:.*: . ..: ..* *:::*.:: .* ::.*  :. *

CCHF            QIVHPQSATPITVQDTHPSPTNRSKRNLKMEIILTLSQGLKKYYGKILRLLQLTLEEDTE
Aigai           QPTTMPNTTALVTPTAHISPTNRSKRNSEVEIILTLPQGLKKYYSKILKLLHLTLEEDSE
                * .   .:*.:..  :* ********* ::******.*******.***:**:******:*

CCHF            GLLEWCKRNLGLDCDDTFFQKRIEEFFITGEGHFNEVLQFRTPGTLSTTESTPAGLPTAE
Aigai           GLLEWCTRTLKQACDDGYFQERIKEFFLTGEGYFNEVLLFRSPSQLGTTGSPLASPPTAE
                ******.*.*   *** :**:**:***:****:***** **:*. *.** *. *. ****

CCHF            PFKSYFAKGFLSIDSGYYSAKCYSGTSNSGLQLINITRHSTRIVDTPGPKITNLKTINCI
Aigai           PFKSFYAKGFLIMDSGYFSAKCYPKASNSGLQLINITQHSIKVVNTPGPKYSNLKTVNCI
                ****::***** :****:*****. :***********:** ::*:***** :****:***

CCHF            NLKASIFKEHREVEINVLLPQVAVNLSNCHVVIKSHVCDYSLDIDGAVRLPHIYHEGVFI
Aigai           NLKVSTDKDHSELEVNVLLPQVAVNISNCHVLIKSHVCDYSLDTDGMIRLPQVTHEGTFI
                ***.*  *:* *:*:**********:*****:*********** ** :***:: ***.**

CCHF            PGTYKIVIDKKNKLNDRCTLFTDCVIKGREVRKGQSVLRQYKTEIRIGKASTGS
Aigai           PGTYKIVIDKKNKLNDRCTMTTNCVIKGKEVRKGQSTLRQYRTEIRVGQMSSGT
                *******************: *:*****:*******.****:****:*: *:*:
```

Figure S1: Expression of GP38/85 mutants and GP85 proteins homology

*A-B) All constructs were produced by transient transfection of HEK293T cells. The soluble proteins in supernatants were purified by affinity chromatography on Co^{2+} resins. C) Binding of GP38/85 vs an unrelated protein (VSV-N) onto HEK293T cells, analysis by flow cytometry D) Alignment of GP85 sequences from CCHF IBAR10200 and Aigai virus using MAFFT. * identical sequence, : conservative mutation, . semi conservative mutation.*

Supplementary figure 2: Computer simulations - Molecular dynamics simulations.

Molecular Dynamics (MD) simulations were carried in the presence of explicit solvent and a HS hexamer as a GP38 ligand. To enhance sampling, four initial orientations of HS hexamer in respect to the binding site were considered. In systems 1 and 2, the heparin chain was oriented in two opposite directions as follows from the direction of the chain raising from non-reducing to reducing end. In systems 3 and 4 the heparin chain was rotated 180° along its transverse axis in respect to their initial positions in 1 and 2, respectively, this manipulation changed the orientation of sulfate and uronic groups to the opposite. On the other hand, such rotation mimicked a lateral shift of the chain by two sugar residues along the binding site. The root-mean-square deviation of HS and the visual analysis of MD trajectories revealed that within the first 100 ns the ligand was exploring the protein surface with an eventual formation of stable complexes (supplementary figure S2A and S2B). The equilibrated complexes were sorted by binding energy ($\Delta G_{binding} = \Delta G_{complex} - \Delta G_{receptor} - \Delta G_{ligand}$). In the most energy-favorable complex with HS (system 1), the NS group belonging to one of N-sulfated glucosamine residues was directed to K471 and K474. A similar complex was formed starting with system 3, the equilibrated polysaccharide was shifted from the initial position on the distance of two sugar residues to set up the contacts between NS and K474/K471, as in system 1. The per-residue analysis of protein-polysaccharide contact population revealed that the residues K409, K396, N394, R412, K471, K472, R478, R507 (see full list in table S1) were among the most tightly interacting with HS along the trajectories.

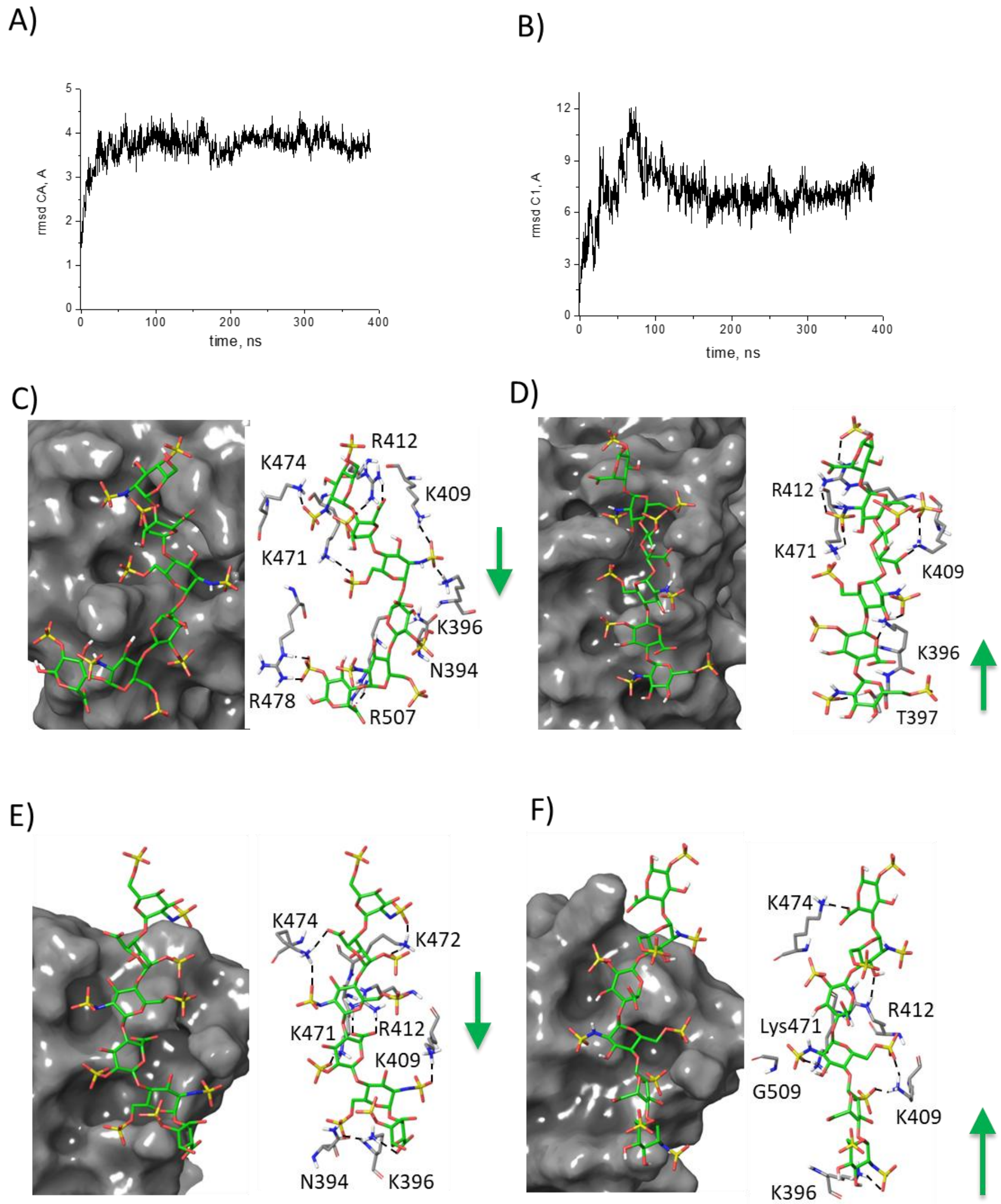

Figure S2. *(A) Root-mean square deviation of Cα-atoms of GP38 in the course of MD trajectory. (B) Root-mean square deviation of C1-atoms of HS in the course of MD trajectory. (C) Structure of system 1 (ΔG = -65 ± 12 kcal/mol), (D) system 2 (ΔG = -51 ± 11 kcal/mol), (E) system 3 (ΔG = -47 ± 14 kcal/mol), (F) system 4 (ΔG = -56 ± 12 kcal/mol) equilibrated in the course of MD trajectory. Left: protein surface view, ligand shown as stick, Right: Residues having close contacts to HS. Color code: C atoms – grey for protein and green for heparin sulfate, O atoms – red, N – nitrogen, S – yellow, polar H – white, non-polar H are not shown for clarity. Arrows indicate the direction from non-reducing to reducing end of HS.*

Supplementary figure 3 : GP38-Gn-GC complex

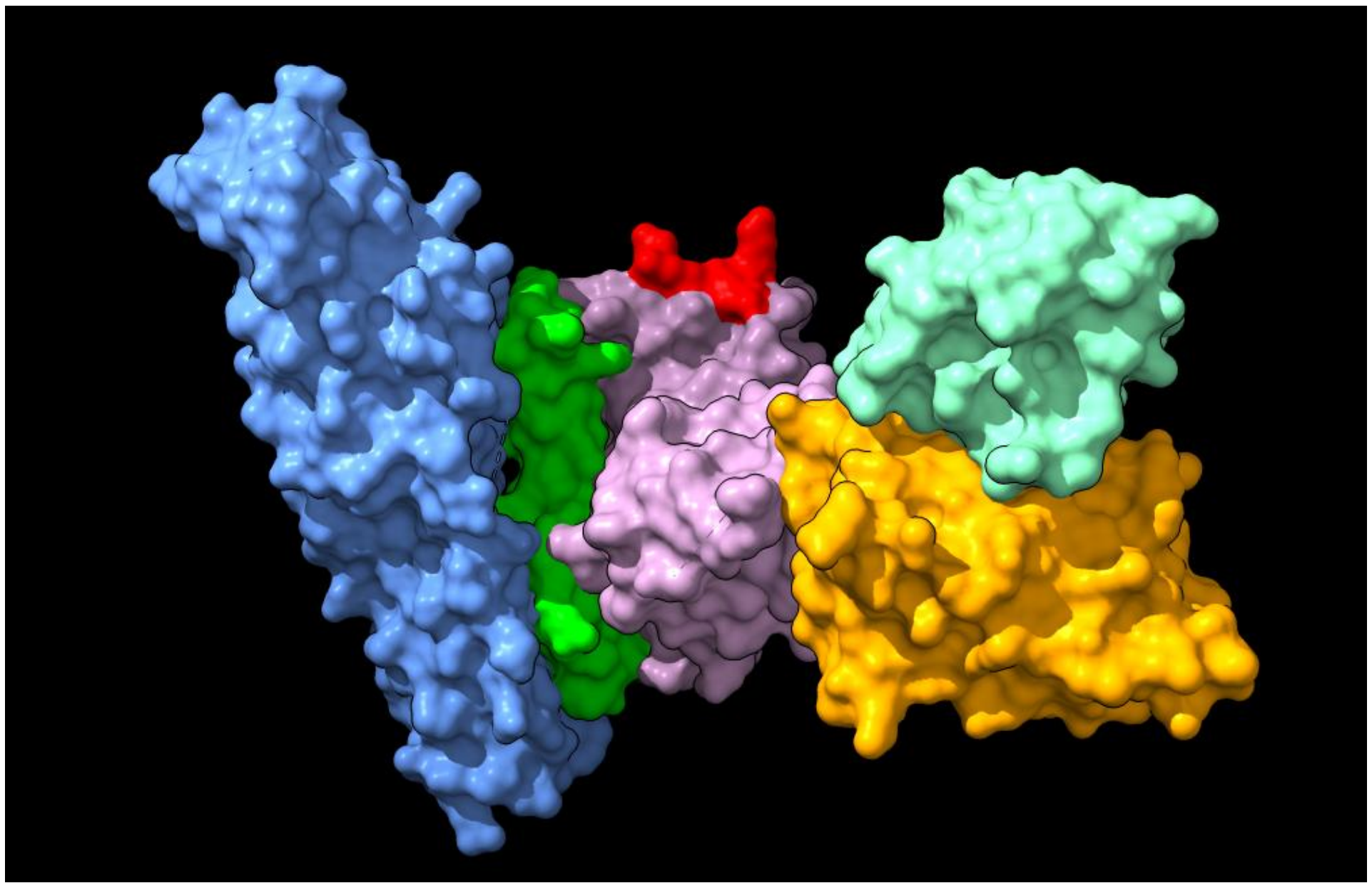

Figure S3: GAG biding domain in the GP38-Gn-Gc complex

The GAG-binding domain was mapped on the structure of GP38-Gn-Gc -Mab ADI46152 (PDB 9B8J) using Chimera software. Cardin-Weintraub motif and Arginine 412 are colored in red, GP38 in magenta, Gc in blue, Gn in green and Mab ADI-46152 lighchain in light green, heavy chain in orange.

Supplementary figure 3 : differential GAGs level between cell lines

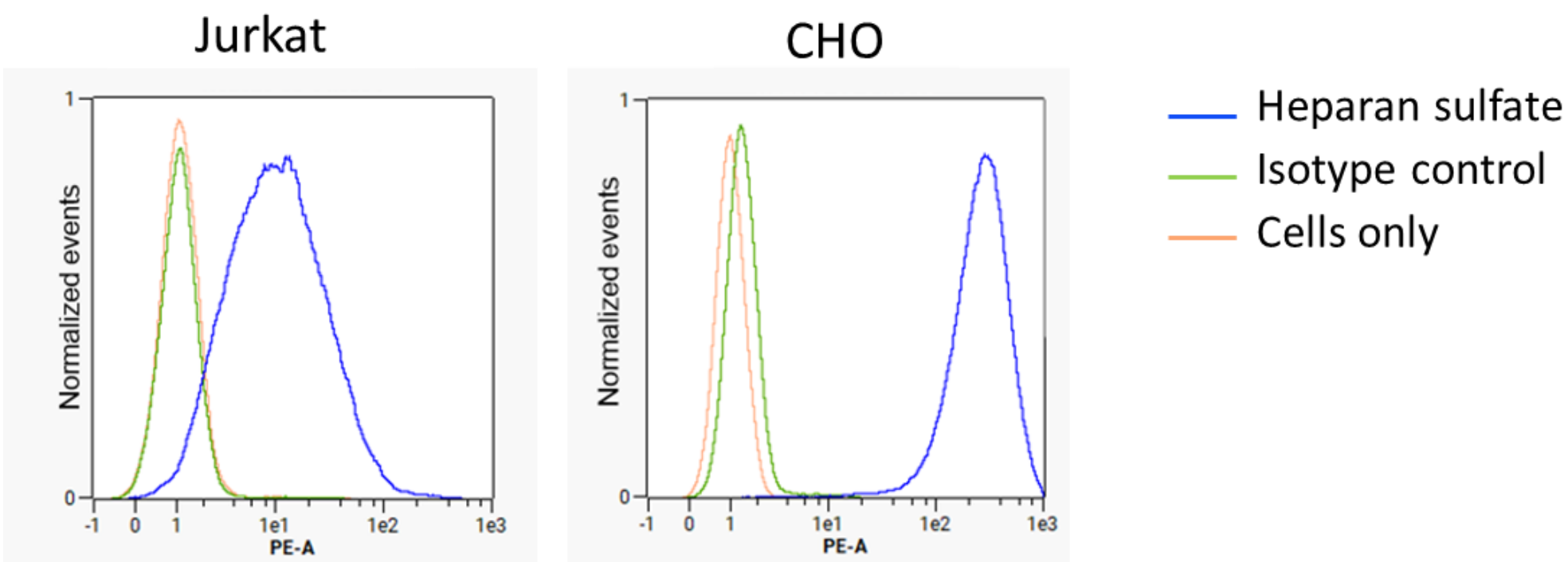

Figure S4 : Differential HS expression in CHO and Jurkat cell line

Cell surface staining of Jurkat cells (Left) and CHO cells (right) with clone 10E4 anti heparan sulfate antibody and secondary anti mouse Cy3 conjugated antibody.

Table S1. Residues with closes contacts with HS in the course of MD trajectories (cut-off 0.6 nm)

	Distance, nm	Standard deviation, nm
System 1		
Arg412	0.184	0.012
Arg507	0.192	0.050
Lys471	0.197	0.023
Lys396	0.219	0.070
Gly509	0.240	0.020
Lys409	0.241	0.095
Asn394	0.244	0.057
Ile508	0.357	0.058
Ile407	0.361	0.107
Asp477	0.378	0.071
Ile392	0.394	0.095
Arg478	0.402	0.149
Lys472	0.418	0.084
Leu395	0.454	0.117
Asn476	0.517	0.077
Lys474	0.537	0.186
System2		
Arg412	0.187	0.023
Lys471	0.192	0.023

Lys409	0.206	0.054
Lys396	0.207	0.067
Asn394	0.265	0.134
Gly509	0.274	0.026
Lys474	0.371	0.203
Asp477	0.375	0.057
Ile508	0.404	0.051
Lys472	0.412	0.127
Ile407	0.458	0.118
Arg507	0.462	0.175
Leu395	0.503	0.122
Glu410	0.525	0.150
Arg478	0.543	0.039
System3		
Arg412	0.189	0.027
Lys396	0.213	0.067
Lys471	0.248	0.092
Lys409	0.253	0.115
Asn394	0.269	0.129
Lys472	0.271	0.101
Gly509	0.308	0.115
Lys474	0.335	0.193
Ile508	0.432	0.124
Ile407	0.461	0.128
Leu395	0.490	0.117
Asp477	0.504	0.139
Arg507	0.537	0.133
System4		
Arg412	0.18	0.020
Lys471	0.200	0.021
Lys396	0.212	0.057
Lys409	0.234	0.093
Asn476	0.287	0.121
Gly509	0.330	0.097
Lys472	0.331	0.116
Ile407	0.349	0.124
Lys474	0.374	0.179
Asn394	0.437	0.213
Ile508	0.486	0.064
